# Introduction of Hungarian Association of Psychiatric Trainees - EPA - Hungarian NPA Joint Symposium

**DOI:** 10.1192/j.eurpsy.2024.1727

**Published:** 2024-08-27

**Authors:** F. Kupcsik

**Affiliations:** Psychiatry and Psychotherapy, Semmelweis University, Budapest, Hungary

## Abstract

**Introduction:**

The purpose of my presentation is to introduce the Hungarian Association of Psychiatric Trainees (HAPT), - our NAT - to you, which includes residents and young specialists within five years of training.

**Objectives:**

Currently we have 108 members, from 15 cities and villages throughout Hungary, and one person is working in Denmark. The vast majority (58 %) of the members are from Budapest, our capital city. There are 14 members, who are young specialists, the others are doing residency training. We have 21 members who are working in child and adolescent psychiatry.

HAPT has been existed since 2013, so in the previous years, our founder members have reached the point when they no longer meet the criteria of being ‘psychiatric trainee’ or ‘young specialists’, however every year we encourage the new residents to join us.

**Methods:**

-

**Results:**

**OUR DUTY:** The main goals of HAPT are educating ourselves, forming a community and making connections with colleagues country-wide and last but not least, trying to stand up for our interests, when needed.

Throughout the year we organize educative presentations about topics that are somehow left out of focus during the official training program. Every year our main event is a three-day long weekend, where we can go deeper into a couple of topics via presentations or workshops, and it is also a great opportunity to get to know each other better.

We also organize case-discussion-groups according to the Balint method, considering the residents’ daily difficulties and trying to pay more attention to their mental well-being.

Last year we tried some other ways to broaden our perspectives in the form of cultural events, when we watched a movie or a play and then discussed it together as a group, had been led by a psychotherapist.

HAPT is part of the Hungarian Psychiatric Association and the relationship between the two Organizations has a constantly changing dynamics – in some ways we are trying to be more independent, however, there are common goals that are important for all of us, for example being present on at international events.

**Conclusions:**

**FUTURE GOALS:** One of our future plans include being more active in the European community, like getting to know the EFPT or the ECP better. This conference is a perfect opportunity for all of us to make new professional connections.

**Disclosure of Interest:**

None Declared

